# Reduced weighted permutation entropy in visual-temporal cortex and altered resting-state brain dynamics in Alzheimer’s disease

**DOI:** 10.3389/fnagi.2026.1921894

**Published:** 2026-10-22

**Authors:** Wenjing Zhao, Yuhong Huang, Hongyi Li, Wei Jian, Ziqi Wang, Tingting Wang, Xue Qiu, Xuemei Chen, Shicai Li, Man Liu, Hui He

**Affiliations:** The Clinical Hospital of Chengdu Brain Science Institute, University of Electronic Science and Technology of China, Chengdu, Sichuan, China

**Keywords:** Alzheimer’s disease, brain complexity, dorsal visual pathway, functional connectivity, resting-state fMRI, weighted permutation entropy

## Abstract

**Background:**

Alzheimer’s disease (AD) is increasingly recognized as a disorder of distributed neural dynamics. Resting-state fMRI combined with signal-complexity measures may provide new insights into alterations in spontaneous brain activity. This study investigated whether weighted permutation entropy (WPE), an amplitude-sensitive measure of temporal complexity, could identify regional abnormalities in AD and characterize their temporal and network-level features.

**Methods:**

Twenty patients with AD and twenty-four healthy controls underwent resting-state fMRI. Regional WPE was calculated using the Brainnetome atlas. Group comparisons included age, sex, years of education, and mean framewise displacement as covariates. Brainnetome findings were evaluated at an exploratory uncorrected threshold of *p* < 0.001 and checked against Bonferroni correction across 246 regions. Selected regions were used for secondary seed-based functional connectivity analysis. Four complexity-drop cascade intervals were compared without multiplicity correction. Sensitivity analyses examined window length, AAL parcellation, and cascade recruitment thresholds of 8, 10, and 12 regions; Fourier-surrogate analyses provided an additional model-based assessment.

**Results:**

At the exploratory Brainnetome threshold, AD was associated with lower WPE in two right inferior temporal subregions and one left fusiform subregion; these findings did not survive Bonferroni correction across 246 regions. The AAL analysis showed a similar regional pattern under FDR correction (*q* < 0.05). Secondary seed-based analyses identified increased connectivity with visual, parietal, and sensorimotor regions. Longer Peak-to-End and Start-to-End cascade intervals were observed in AD at nominal *p* < 0.05, without correction across the four interval tests. The overall pattern of subsequent cascade analyses was retained at the 8- and 12-region thresholds.

**Conclusion:**

This small-sample study suggests that AD is associated with regional WPE differences, altered connectivity, and possible prolongation of complexity-drop cascades. The exploratory Brainnetome and temporal findings, together with within-sample AAL convergence, motivate independent replication with prespecified multiplicity control. These measures do not yet establish a clinical biomarker or a specific physiological mechanism.

## 1 Introduction

Alzheimer’s disease is the most common neurodegenerative cause of dementia and is classically characterized by progressive memory impairment, executive dysfunction, and deterioration of daily functioning (Saykin and Rabin, 2014; Sohrabi and Weinborn, 2019). Although the molecular pathology of AD has been linked to amyloid-beta deposition, tau pathology, synaptic dysfunction, and neurodegeneration (Spires-Jones and Hyman, 2014; Li et al., 2018; John and Reddy, 2021; Abyadeh et al., 2024), the clinical expression of the disease depends strongly on how these processes disrupt large-scale brain activity. Neuroimaging studies have therefore moved beyond the search for isolated structural abnormalities and increasingly examine AD as a disorder of distributed functional systems (Dickerson and Sperling, 2009; Reid and Evans, 2013; Jagust, 2018; Jones et al., 2016).

Resting-state fMRI has been especially influential in this shift because spontaneous BOLD fluctuations provide information about intrinsic brain organization without requiring explicit task performance (Biswal et al., 1995, 2010). Most AD studies using resting-state fMRI have focused on functional connectivity, particularly the default mode network, hippocampal-cortical interactions, and large-scale disconnection (Ewers et al., 2021; Penalba-Sánchez et al., 2023; Ghaffari et al., 2025). However, functional connectivity reflects statistical dependence between regions and does not directly quantify the temporal complexity of each regional signal. A region may show abnormal local activity dynamics even before or independently of connectivity changes. For neurodegenerative disorders, such local dynamics may be important because synaptic failure, impaired excitation-inhibition balance, vascular dysregulation, and reduced adaptive capacity can all alter the temporal structure of BOLD signals (Burté et al., 2015).

Signal complexity offers a complementary framework for studying these changes. Entropy-based measures quantify the richness, irregularity, or diversity of temporal patterns in neural signals (Krohn et al., 2023). In the context of BOLD fMRI, higher complexity is often interpreted as a greater capacity to explore dynamic states, whereas lower complexity may indicate more stereotyped and less flexible neural activity. Importantly, resting-state BOLD signal variability is also altered across the AD spectrum, with previous studies reporting spatially and frequency-dependent decreases or increases rather than a uniform global reduction (Zhang et al., 2020). Moreover, previous rs-fMRI studies have already provided evidence that brain signal complexity is altered across the Alzheimer’s disease spectrum. Using permutation entropy, Wang et al. (2017) found lower resting-state BOLD complexity in AD than in MCI and healthy controls, with significant abnormalities predominantly involving occipital, temporal, and frontal regions. Earlier work using approximate entropy also demonstrated reduced resting-state BOLD complexity with aging and cognitive decline associated with familial AD (Liu et al., 2013). Similarly, an amplitude-aware permutation entropy study in individuals with subjective cognitive decline identified reduced complexity in several association regions, including the superior temporal cortex (Ni et al., 2021). These findings suggest that disruption of spontaneous BOLD complexity may emerge across different stages of the AD spectrum, although the precise spatial distribution varies with the entropy metric, disease stage, and analytical framework. However, most previous AD-related entropy studies have primarily characterized regional or static complexity differences. Whether AD also alters the temporal organization and persistence of coordinated complexity reductions remains less well understood.

Within this framework, classical permutation entropy characterizes the diversity of ordinal temporal patterns, whereas weighted permutation entropy (WPE) additionally weights these patterns according to their amplitude variation (Fadlallah et al., 2013). WPE therefore reflects both temporal pattern diversity and the distribution of amplitude information across these patterns. Importantly, WPE incorporates relative rather than absolute amplitude variation and is invariant to linear transformations of signal amplitude (Krohn et al., 2023). Moreover, previous simulations and empirical BOLD analyses have demonstrated that WPE can vary despite unchanged signal variance and can distinguish BOLD dynamics that are not captured by signal variance or permutation entropy alone (Krohn et al., 2023). The spatiotemporal complexity architecture described by Krohn et al. (2023) further demonstrated that WPE can characterize both regional complexity and transient complexity drops in human brain activity. These properties make WPE particularly suitable for examining whether AD is associated not only with altered BOLD fluctuation magnitude, but also with abnormalities in the temporal organization and flexibility of spontaneous brain activity.

Building on the WPE-based spatiotemporal complexity framework established in previous work, the present study investigated whether the local, temporal, and network organization of spontaneous BOLD complexity is altered in AD. We first examined regional WPE abnormalities, then characterized the network-level connectivity patterns associated with WPE-altered regions, and finally quantified the temporal persistence of complexity-drop cascades using predefined temporal landmarks. Rather than proposing new categorical brain states, our aim was to determine whether AD modifies an established spatiotemporal complexity architecture and to relate regional complexity loss to its temporal and network-level characteristics.

## 2 Materials and methods

### 2.1 Participants

The study included 20 patients with AD and 24 healthy controls recruited from the Clinical Hospital of Chengdu Brain Science Institute. Diagnosis of dementia due to Alzheimer’s disease was established according to the NIA-AA 2011 clinical criteria for probable AD dementia (McKhann et al., 2011) and the DSM-5 criteria for major neurocognitive disorder due to Alzheimer’s disease. Healthy controls were clinically screened as cognitively normal and had no history of neurological or psychiatric disorders or clinically significant cognitive impairment. Inclusion criteria for patients with AD were a clinical diagnosis of AD, the ability to complete MRI scanning, and analyzable resting-state fMRI data. Exclusion criteria included major stroke, other neurodegenerative disorders, severe psychiatric illness, clinically significant brain lesions, and contraindications to MRI; these criteria were also applied to healthy controls where relevant. Clinical characterization of the AD group included disease duration, Mini-Mental State Examination (MMSE), Montreal Cognitive Assessment (MoCA), Activities of Daily Living (ADL), and available medication information, including medication type and daily dose. Individual-level clinical data are provided in Supplementary Table 1. Quantitative cognitive scores were not available for the healthy control group.

### 2.2 MRI data acquisition

MRI data were acquired using a 3.0 T MRI scanner (Siemens, Erlangen, Germany). Resting-state fMRI images were obtained using a gradient-echo echo-planar imaging (EPI) sequence (repetition time: 2000 ms, echo time: 30 ms, flip angle: 90°, matrix size: 64 × 64, field of view: 240mm × 240 mm, slice thickness/gap: 4/0.4 mm). Each patient completed a 510-s resting-state scan, yielding a total of 255 volumes. Resting-state fMRI data were collected while participants were instructed to keep their eyes closed, remain awake, avoid structured thinking, and minimize head movement. High-resolution T1-weighted anatomical images were acquired using a 3-dimensional fast spoiled gradient echo (T1-3D FSPGR) sequence: TR = 2300 ms, TE = 2.32 ms, FA = 8°, matrix = 256 × 256, slice thickness = 0.9 mm, 192 slices without a gap.

### 2.3 Preprocessing of resting-state fMRI

Functional image preprocessing was performed in DPABI^1^ (Yan et al., 2016). The preprocessing pipeline included removal of initial volumes, slice-timing correction, realignment for head-motion correction, coregistration with anatomical images, spatial normalization, and spatial smoothing with a 6 mm FWHM Gaussian kernel. Nuisance regression incorporated Friston-24 motion parameters, white matter and cerebrospinal fluid signals, and linear trends. Volumes exhibiting framewise displacement >0.2 mm were treated as outliers and included as separate regressors. Global signal regression was not performed. The fMRI data were filtered within a frequency range of 0.01–0.08 Hz. Participants with excessive head motion were excluded using predefined criteria, such as translation greater than 2 mm, rotation greater than 2 degrees, or mean framewise displacement (FD) greater than 0.2.

### 2.4 Weighted permutation entropy analysis

Weighted permutation entropy is an amplitude-sensitive variant of permutation entropy that retains informative amplitude-related characteristics of the signal. As illustrated in Figure 1A, for each participant, BOLD time series were extracted using the Brainnetome (BN) atlas (Fan et al., 2016), which includes 246 cortical and subcortical regions of interest. Following prior WPE-fMRI studies (Krohn et al., 2023; Liu et al., 2024), WPE was computed with an embedding dimension (motif length) of *m* = 3 and a time delay of τ = 1. Within each sliding window, ordinal motifs were identified from the regional BOLD signal, and the occurrence frequency of each motif was weighted by the local amplitude variance associated with that motif. The variance-weighted motif frequencies were normalized to sum to one, and WPE was calculated as the Shannon entropy of this weighted distribution and normalized by log(m!) to yield values between 0 and 1. The regional BOLD time series were partitioned into overlapping windows of 20 TRs (40 s, 95% overlap), which approximates the 43.2-s physical window used by Krohn et al. (2023) while accounting for the longer TR (2.0 s) of the present acquisition. Window-level WPE values were calculated for every ROI, and the mean WPE across all windows was used as the participant-level regional WPE measure for group comparison. For the primary Brainnetome-based analysis, the group effect on WPE was evaluated using a general linear model with age, sex, years of education, and mean FD entered as covariates. The original BN screen used uncorrected *p* < 0.001 across 246 ROIs. To make the multiplicity limitation explicit, we additionally evaluated the reported candidate regions using Bonferroni-adjusted *p*-values, calculated as min (246 × p, 1). The uncorrected regional findings are treated as exploratory. FDR correction for the AAL analysis is described in sensitivity analyses section.

**FIGURE 1:**
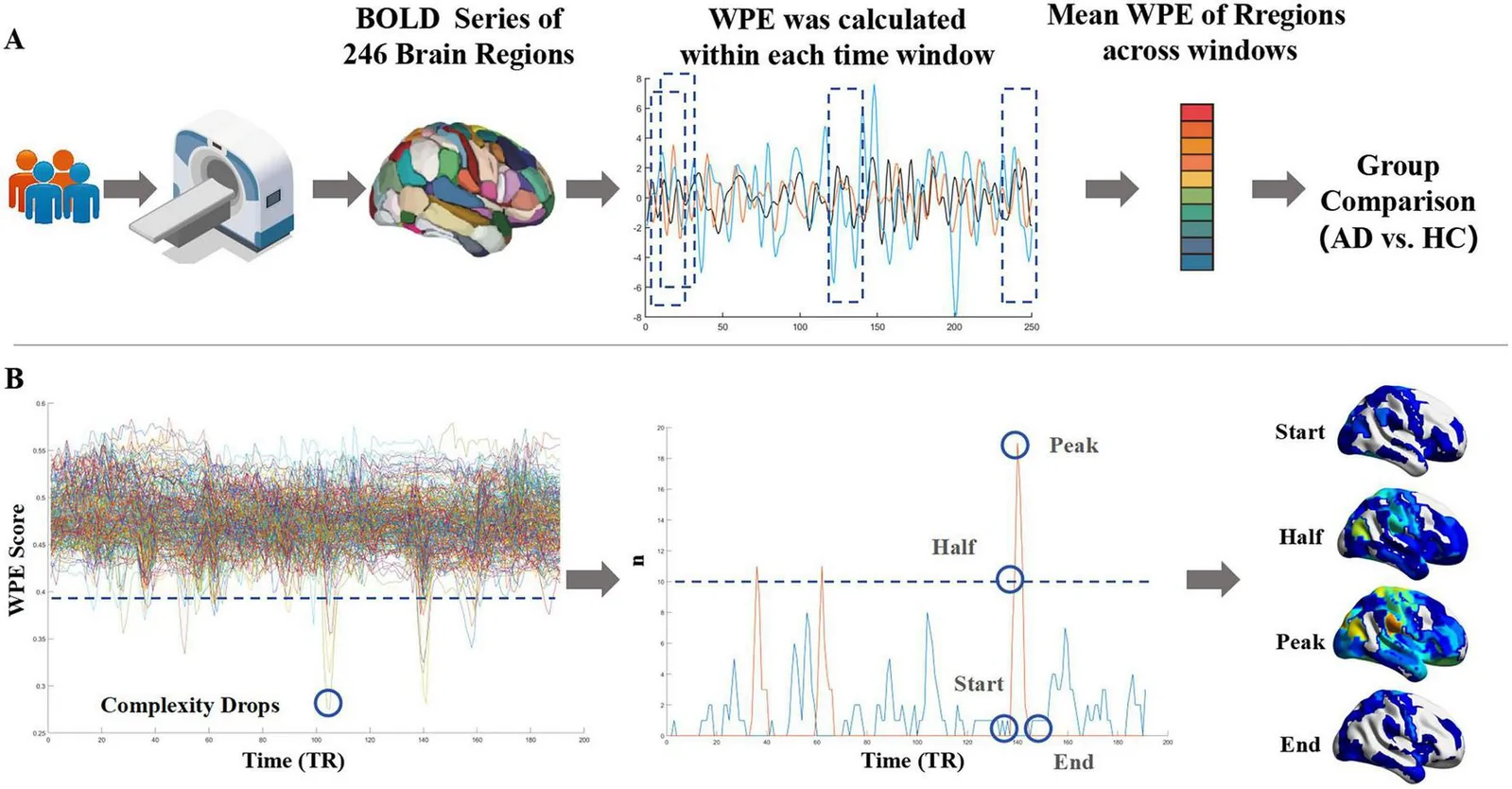
Study workflow. **(A)** Resting-state fMRI data were acquired from patients with AD and healthy controls, preprocessed, parcellated into 246 ROIs, and analyzed using regional WPE. **(B)** Subject-level WPE sequences were further used to characterize complexity-drop cascades and quantify four temporal landmarks–Start, Half, Peak, and End–and the corresponding cascade-duration metrics.

### 2.5 Seed-based functional connectivity analysis

Brain regions showing significant between-group WPE differences were subsequently used as seeds to characterize their network-level connectivity patterns. The seed regions corresponded to predefined anatomical parcels from the Brainnetome atlas. This seed-based FC analysis was considered a secondary network-characterization analysis rather than an independent statistical validation of the regional WPE findings. For each seed and each participant, Pearson correlation coefficients were calculated between the seed regional BOLD time series and the time series of all other voxels, and correlation coefficients were transformed to Fisher z scores before statistical comparison. Between-group FC differences were assessed in a general linear model including age, sex, years of education, and mean FD as covariates. Multiple comparisons were controlled using Gaussian random field (GRF) correction with a two-tailed voxel-level threshold of *p* < 0.005 and a cluster-level threshold of *p* < 0.05.

### 2.6 Temporal organization of complexity- drop cascades

As illustrated in Figure 1B, the WPE matrix was represented as a temporal profile characterized by predominantly high-complexity activity interspersed with transient reductions in complexity. The following measures were defined as operational descriptors of WPE dynamics. Consistent with the original spatiotemporal WPE framework of Krohn et al. (2023), a complexity drop was identified when the WPE value of a brain region fell below the first percentile of the overall WPE distribution. For each time window, the number of brain regions exhibiting a complexity drop was counted, generating a temporal curve in which the *x*-axis represented successive time windows and the *y*-axis represented the number of regions showing reduced complexity.

To characterize the temporal organization and spatial recruitment of these complexity reductions, we identified complexity-drop cascades, as illustrated by the blue lines in Figure 1B. Following the propagation implementation used in prior WPE work, the primary cascade definition required at least 10 brain regions to exhibit a complexity drop simultaneously within a BOLD window. When multiple consecutive windows met this criterion, the window containing the largest number of affected regions was defined as the peak of the cascade. Starting from this peak, the cascade was traced both backward and forward in time through neighboring windows in which the number of affected regions progressively decreased. The tracing procedure was terminated when the number of affected regions remained unchanged or increased.

To characterize the temporal evolution of each cascade, four temporal landmarks–Start, Half, Peak, and End–were identified along the cascade trajectory. These landmarks were used solely to describe the progression of complexity-drop events and should not be interpreted as categorical biological brain states. WPE vectors were extracted at each landmark to characterize the spatial distribution of complexity reductions. For each landmark, the probability of involvement of each brain region was calculated from the frequency with which that region exhibited a complexity drop at the corresponding cascade window. For each participant, cascade-duration metrics were quantified as the intervals between temporal landmarks, including Start-to-Half, Half-to-Peak, Peak-to-End, and Start-to-End. Age, sex, years of education, and mean FD were included as covariates in the group comparison. Because these duration measures were not assumed to follow a normal distribution, between-group differences were assessed using covariate-adjusted permutation testing. No multiple-comparison correction was applied across the four interval measures in the original analysis; therefore, these temporal comparisons are interpreted as exploratory and nominal *p*-values are reported. For temporal measures showing significant group differences, effect sizes were quantified using Hedges’ g, and 95% confidence intervals were estimated using bootstrap resampling.

### 2.7 Fourier-based temporal surrogate validation

To determine whether the observed WPE abnormalities and drop cascades could be explained by the intrinsic temporal autocorrelation of the BOLD signal, we additionally performed a Fourier phase-randomized surrogate analysis. One thousand null data sets were generated using a Fourier-based surrogate method for each regional BOLD time series to estimate the statistic’s distribution under the null hypothesis. This procedure preserves the amplitude/power spectrum, and consequently the linear autocorrelation properties of the original BOLD signal, while disrupting its original temporal phase organization (Prichard and Theiler, 1994).

Detailed steps were described as follows (Supplementary Figure 1A): (1) The discrete Fourier transform was applied to *X*_1_, *X*_2_, …,*X_n_* of one BOLD signal x. (2) A random phase: $\tilde{X}$n = *X*_*n*_*e*^*i*φ_*n*_^, where *φ*_1_, …, *φ*_*n*_ is a vector of independent stochastic variables that are uniformly distributed in the interval [-π, π]. (3) The inverse discrete Fourier transformation was applied to $\tilde{X}$n to yield randomized $\tilde{x}$ of x. (4) For each surrogate realization, the complete WPE calculation and drop-cascade analysis were repeated using the same parameters as those applied to the original data. (5) Two complementary empirical null tests were performed. First, for each region showing a significant between-group WPE difference in the original analysis, the AD–HC WPE difference was recalculated from each surrogate dataset, resulting in a null distribution of 1,000 between-group differences. The observed WPE group difference was then compared with this surrogate distribution to obtain an empirical probability. Second, the number of drop cascades was calculated for each participant in each surrogate dataset using the same cascade definition as in the original analysis. The corresponding group-level AD-HC cascade difference was calculated for each surrogate realization to construct a null distribution, against which the observed score derived from the original BOLD data was evaluated (Hindriks et al., 2016).

### 2.8 Window-length, atlas and cascade- definition threshold sensitivity analyses

To evaluate whether the WPE findings were dependent on the selected sliding-window length or brain parcellation scheme, we performed two additional sensitivity analyses. First, WPE was recalculated using window lengths of 10, 20, and 30 TRs, corresponding to 20, 40, and 60 s, respectively. For each window length, WPE was recalculated using the same preprocessing, overlap, and statistical procedures as in the primary analysis, and between-group differences were reassessed.

Second, we evaluated the sensitivity of the complexity-drop cascade definition to the percentile threshold and the minimum number of simultaneously involved regions. To test the robustness of these operational choices, the analysis was repeated using second- and third-percentile thresholds and minimum regional extents of 8 and 12 regions, resulting in four alternative parameter combinations: 2nd percentile/8 regions, 2nd percentile/12 regions, 3rd percentile/8 regions, and 3rd percentile/12 regions. For each combination, cascades were reidentified, and the Start-to-Half, Half-to-Peak, Peak-to-End, and Start-to-End intervals were recalculated for each participant. Group comparisons were then repeated using the same statistical procedures as in the primary analysis.

Third, to examine whether the regional WPE findings depended on the choice of brain atlas, the analysis was repeated using the Automated Anatomical Labeling (AAL) atlas (Tzourio-Mazoyer et al., 2002) instead of the Brainnetome atlas. Regional BOLD time series were re-extracted according to the AAL parcellation, and WPE was recalculated using the same preprocessing, window parameters, and statistical procedures as in the primary analysis. For the AAL-based sensitivity analysis, multiple comparisons across atlas regions were controlled using the Benjamini–Hochberg false discovery rate procedure, with significance defined as FDR-corrected *q* < 0.05. The resulting between-group spatial pattern was then compared with that obtained using the Brainnetome atlas.

## 3 Results

### 3.1 Demographic and clinical characteristics

The final sample included 20 patients with AD and 24 healthy controls. Demographic information and available clinical characteristics of the AD group, including disease duration, MMSE, MoCA and ADL are summarized in Table 1. No significant between-group differences were observed in age, sex, or years of education. Individual-level disease duration, MMSE, MoCA, ADL, medication type, and daily dose for patients with AD are provided in Supplementary Table 1. Quantitative cognitive scores were not available for the healthy control group.

**TABLE 1:** Participant fundamental information.

Demographic variables and clinical characteristics	AD	HC	*P*-value
Gender (female/male)	14/6	14/10	0.641^a^
Age (year)	69.65 (9.08)	66 (7.28)	0.146^b^
Education (year)	8.30 (4.62)	10.46 (3.75)	0.095^b^
Disease duration (year)	3.85 (1.69)	–	–
MMSE	13.85 (8.18)	–	–
MoCA	9.25 (6.73)	–	–
ADL	39.25 (15.27)	–	–

Indicated values are shown as mean (standard deviation).

*^a^*The *p*-value was calculated by chi-square test.

*^b^*The *p*-value was calculated by independent samples *t*-test. AD, Alzheimer’s disease; HC, healthy controls; MMSE, Mini-Mental State Examination; MoCA, Montreal Cognitive Assessment; ADL, Activities of Daily Living.

### 3.2 Regional WPE differences between AD and healthy controls

At the exploratory Brainnetome threshold of uncorrected *p* < 0.001, whole-brain ROI-level WPE analysis identified three regions with reduced WPE in patients with AD relative to healthy controls after adjustment for age, sex, years of education, and mean FD. These included two subregions of the right inferior temporal gyrus (Figures 2A, 3A) and one subregion of the left fusiform gyrus (Figure 4A). No brain region showed increased WPE in AD under the primary exploratory threshold (Figures 2B, 3B, 4B). The direction of effect indicates that spontaneous BOLD activity in these visual association regions was more temporally regular and less complex in AD. Because the primary BN analysis was uncorrected, these regional findings are interpreted together with the FDR-corrected AAL sensitivity analysis rather than as stand-alone confirmatory results.

**FIGURE 2:**
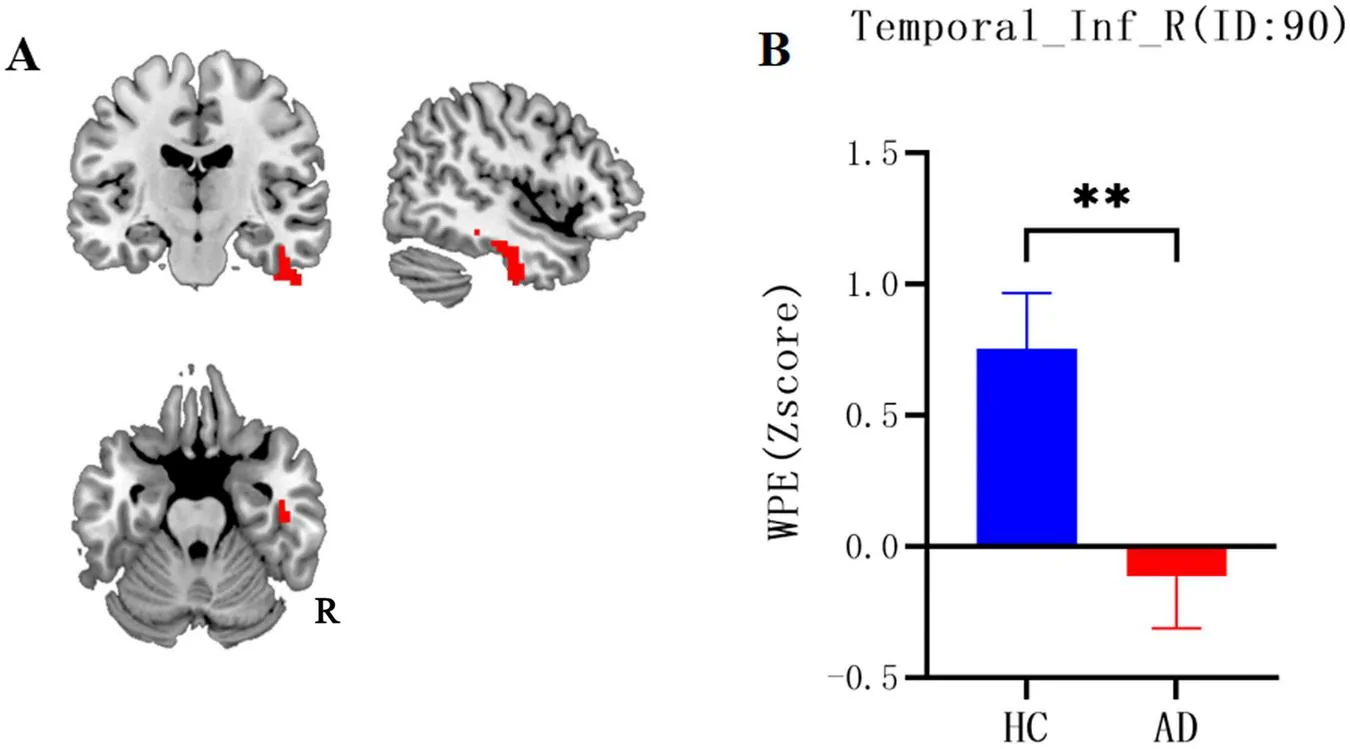
**(A)** Regional WPE difference in the right inferior temporal gyrus (Brainnetome region 90). **(B)** Bar plot of the group difference in WPE. The region was identified at the exploratory Brainnetome threshold of uncorrected *p* < 0.001. “R” indicate the right side of the displayed brain. **nominal *p* < 0.01.

**FIGURE 3:**
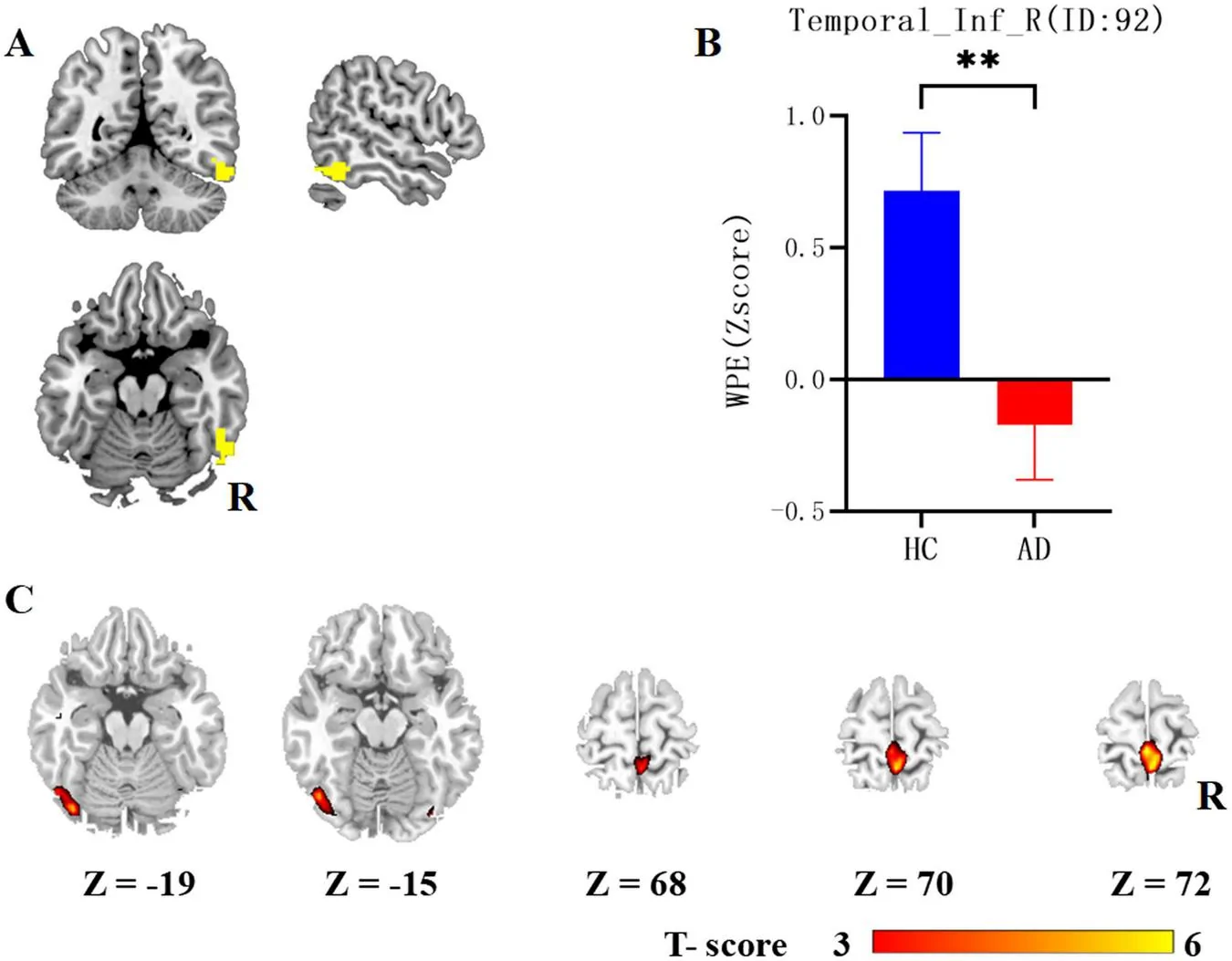
**(A)** Regional WPE difference in the right inferior temporal gyrus (Brainnetome region 92). **(B)** Bar plot of the group difference in WPE. The region was identified at the exploratory Brainnetome threshold of uncorrected *p* < 0.001. **(C)** Seed-based functional connectivity differences between patients with AD and healthy controls. Warm colors indicate AD > HC; two-tailed GRF corrected, voxel *p* < 0.005 and cluster *p* < 0.05. “R” indicate the right side of the displayed brain. **nominal *p* < 0.01.

**FIGURE 4:**
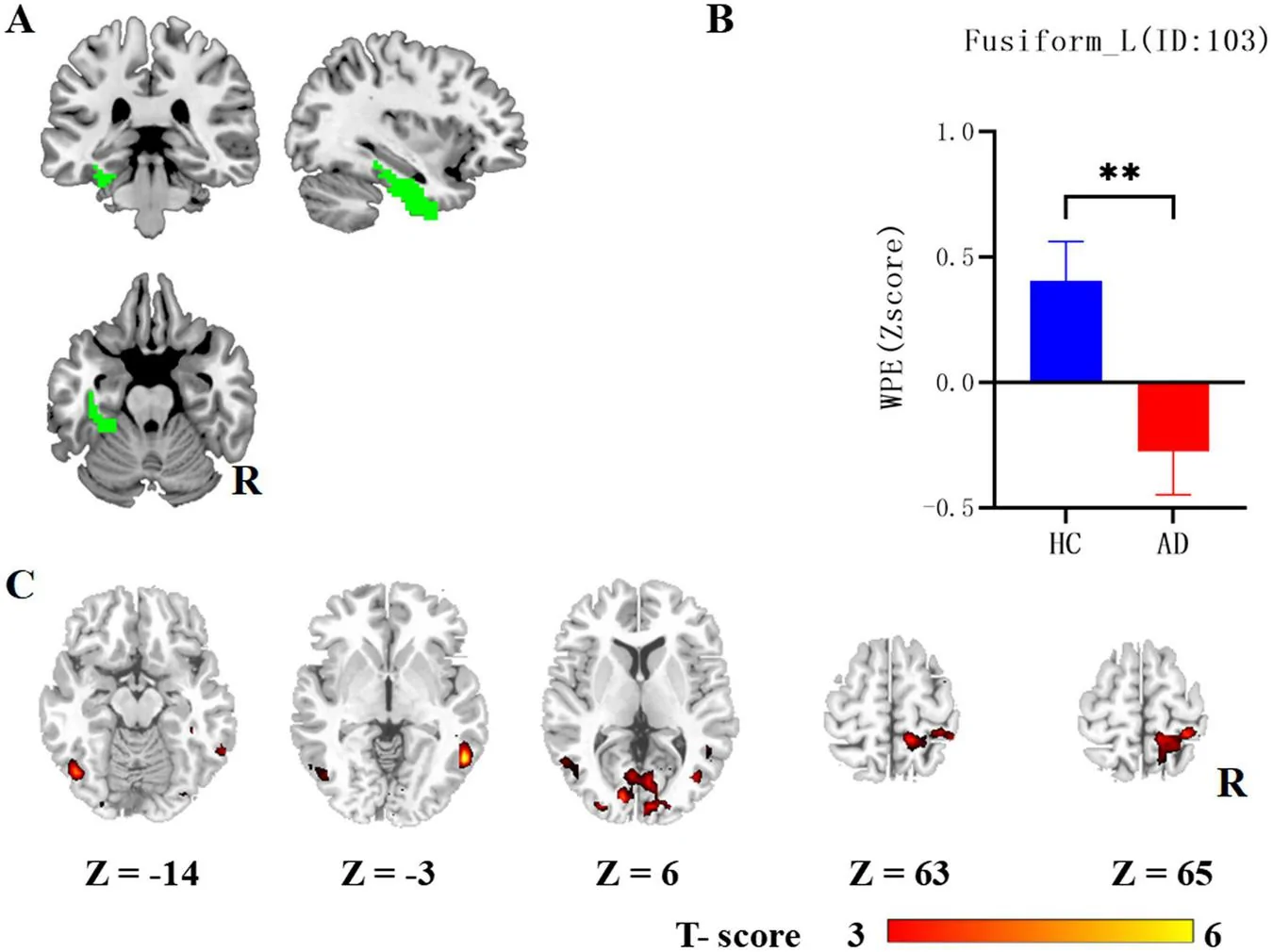
**(A)** Regional WPE difference in the left fusiform gyrus (Brainnetome region 103). **(B)** Bar plot of the group difference in WPE. The region was identified at the exploratory Brainnetome threshold of uncorrected *p* < 0.001. **(C)** Seed-based functional connectivity differences between patients with AD and healthy controls. Warm colors indicate AD > HC; two-tailed GRF corrected, voxel *p* < 0.005 and cluster *p* < 0.05. “R” indicate the right side of the displayed brain. **nominal *p* < 0.01.

### 3.3 Functional connectivity of WPE- altered visual regions

To characterize the network-level connectivity patterns associated with regions showing altered local complexity, the three WPE-altered regions were used as seeds in a secondary seed-based FC analysis. After adjustment for age, sex, years of education, and mean FD, two seed regions–the right inferior temporal gyrus and left fusiform gyrus–showed increased functional connectivity in AD compared with healthy controls under two-tailed GRF correction (voxel *p* < 0.005, cluster *p* < 0.05). The increased connections involved visual cortical and motor/parietal regions associated with the dorsal visual pathway (Figures 3C, 4C and Table 2). These findings are interpreted as complementary network characterization rather than independent validation of the WPE findings.

**TABLE 2:** Significant group differences of seed-based functional connectivity.

Brain region	MNI coordinate (x, y, z)	Cluster voxels	Peak t score
Seed: right inferior temporal gyrus			
Cluster 1			
Right paracentral lobule	0, −39, 72	109	4.94^a^
Right precuneus			
Left paracentral lobule			
Left precuneus			
Seed: left fusiform gyrus			
Cluster 1	12, −87, 3	246	4.22^a^
Left calcarine fissure and surrounding cortex			
Right calcarine fissure and surrounding cortex			
Left lingual gyrus			
Right lingual gyrus			
Cluster 2			
Right inferior temporal gyrus	54, −57, −3	190	5.11^a^
Right middle temporal gyrus			
Cluster 3			
Right postcentral gyrus	9, −54, 75	156	4.85^a^
Right superior parietal gyrus			
Right precuneus			
Cluster 4			
Left middle temporal gyrus	−48, −69, 0	93	3.74^a^
Left middle occipital gyrus			
Left inferior occipital gyrus			
Cluster 5			
Right middle occipital gyrus	42, −69, 12	50	4.06^a^
Right middle temporal gyrus			

*^a^*The *p*-value was calculated by independent samples *t*-test.

### 3.4 Temporal dynamics of complexity- drop cascades

In the primary cascade analysis using the 10-region criterion (Figure 5A), nominal between-group differences were observed in cascade-duration metrics after covariate adjustment. Patients with AD showed a longer Peak-to-End interval than healthy controls (Figure 5B), and the overall Start-to-End duration was also prolonged (Figure 5C). No multiple-comparison correction was applied across the four temporal interval tests; these findings are therefore considered exploratory. For Peak-to-End, patients with AD showed a moderate-to-large effect relative to healthy controls (Hedges’g = 0.781, 95% CI: 0.216–1.360, raw permutation *p* = 0.020). A similar effect was observed for Start-to-End (Hedges’g = 0.762, 95% CI: 0.141–1.530, raw permutation *p* = 0.025).

**FIGURE 5:**
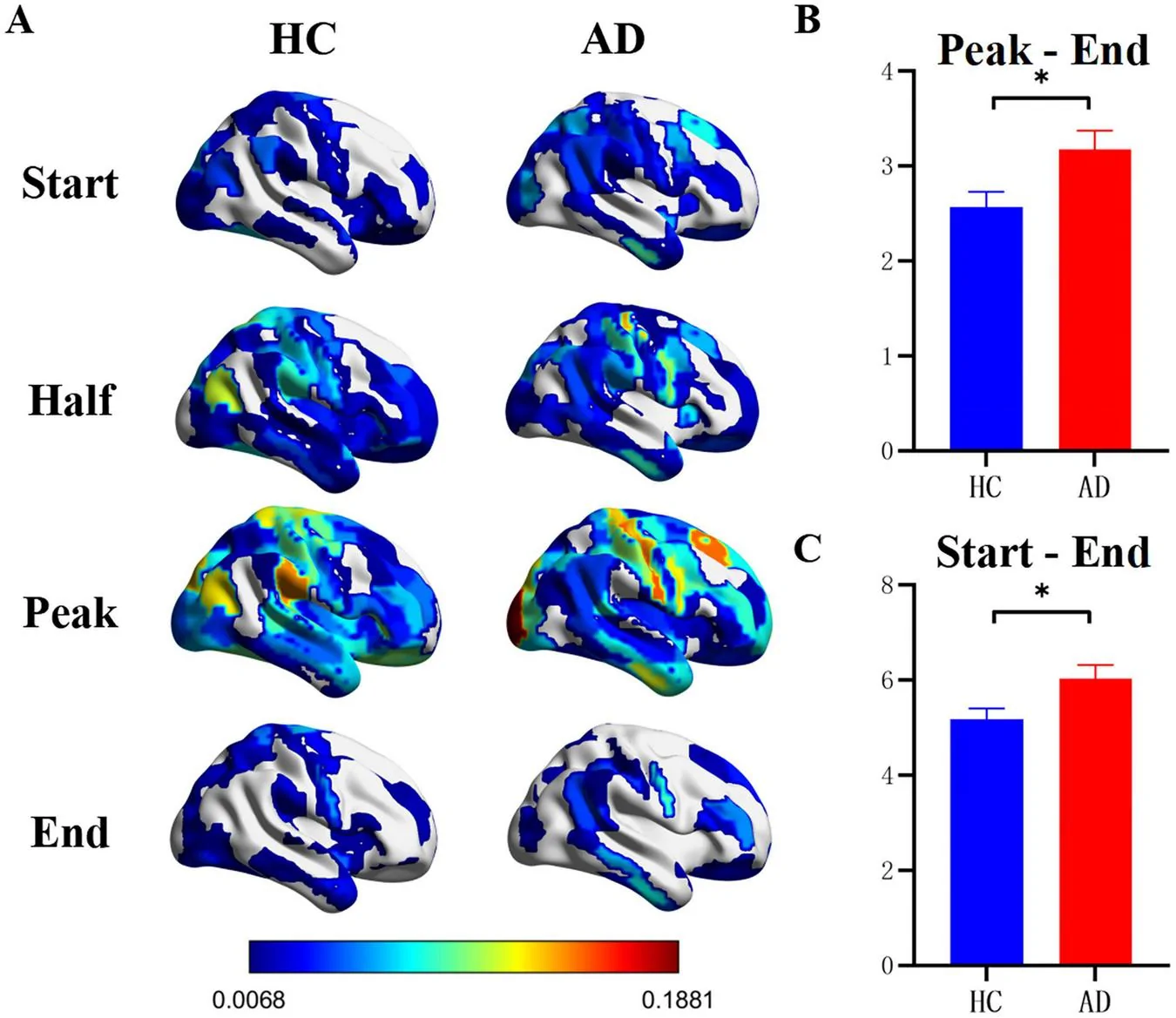
Spatial patterns and temporal characteristics of complexity-drop cascades in Alzheimer’s disease. **(A)** Regional probabilities of involvement in complexity drops at four temporal landmarks (Start, Half, Peak, and End) in healthy controls (HC) and patients with Alzheimer’s disease (AD). Group comparisons of the Peak-to-End **(B)** and Start-to-End **(C)** intervals, respectively. Warmer colors indicate higher probabilities. Patients with AD exhibited longer intervals than HC. *p < 0.05, uncorrected for multiple comparisons. WPE, weighted permutation entropy.

### 3.5 Validation against fourier-based temporal surrogate data

The Fourier-based surrogate analysis confirmed that the main WPE findings were unlikely to result from the intrinsic temporal autocorrelation of the BOLD signal. For each of the three regions showing significantly reduced WPE in AD, the observed between-group WPE difference was compared with the empirical null distribution obtained from 1000 Fourier phase-randomized surrogate datasets. The observed WPE differences were highly unlikely under the surrogate null distributions (Supplementary Figure 1B, *p* < 0.0001). The drop-cascade findings were similarly robust to temporal surrogate testing. The observed group-level cascade difference was compared with the null distribution generated from 1,000 surrogate BOLD signals. The observed cascade difference was highly unlikely under this null model (Supplementary Figure 1C, *p* < 0.001). Together, these surrogate analyses indicate that the regional WPE abnormalities and drop-cascade organization observed in the original data cannot be accounted for solely by the linear autocorrelation structure of spontaneous BOLD fluctuations.

### 3.6 Robustness to window length, brain parcellation, and cascade-definition threshold

The window-length sensitivity analysis showed that the principal pattern of between-group WPE abnormalities was preserved across 10-, 20-, and 30-TR windows. The mean spatial distribution of WPE was also generally consistent across window lengths (Supplementary Figure 2). In addition, the AAL-based sensitivity analysis reproduced a similar visual-temporal pattern, and the corresponding group differences survived FDR correction (*q* < 0.05; Supplementary Table 2). These findings suggest that the principal WPE results were not strongly dependent on the specific choice of sliding-window length or brain parcellation scheme.

The complexity-drop cascade findings were also robust to alternative threshold definitions. When the percentile threshold was varied from the first percentile to the second and third percentiles and the minimum number of simultaneously involved regions was varied from 10 to 8 and 12 regions, the main between-group differences in cascade-duration metrics were reproduced, including the longer Peak-to-End and Start-to-End intervals in AD. The corresponding sensitivity results are provided in Supplementary Table 3.

## 4 Discussion

This study provides a WPE-based characterization of abnormal resting-state brain dynamics in AD. Three main findings emerged. First, patients with AD showed reduced WPE in inferior temporal and fusiform regions, indicating decreased local signal complexity in visual association cortex. Second, seed-based functional connectivity from WPE-altered visual regions revealed increased connectivity between selected seeds and dorsal visual pathway nodes. Third, temporal landmark analysis showed that AD patients had longer Peak-to-End and Start-to-End intervals, indicating prolonged complexity-drop cascades. Together, these findings indicate that AD is associated with abnormalities at three complementary levels: local temporal complexity, network connectivity, and the temporal persistence of complexity-drop cascades.

The regional WPE findings are broadly consistent with previous rs-fMRI complexity studies in the AD spectrum. Wang et al. (2017) examined 29 patients with AD, 33 with early MCI, 32 with late MCI, and 30 normal controls using permutation entropy and found progressively lower complexity toward AD, with significant abnormalities mainly distributed in occipital, temporal, and frontal regions. In particular, their involvement of temporal and occipital cortices is broadly convergent with the inferior temporal and fusiform abnormalities observed in the present study. Consistent abnormalities have also been reported at an earlier stage of the AD spectrum. Using amplitude-aware permutation entropy and the Brainnetome atlas, Ni et al. (2021) found reduced resting-state complexity in subjective cognitive decline, including the superior temporal, inferior parietal, postcentral, and insular subregions. Multiscale entropy studies further demonstrated AD-related complexity abnormalities across temporal scales, including occipital and temporal regions (Niu et al., 2018). These convergent findings support the presence of altered temporal complexity across the AD spectrum, while differences in regional localization may reflect differences in disease stage, entropy formulation, temporal parameters, and parcellation strategy. Accordingly, the novelty of the present findings does not lie in demonstrating reduced brain complexity *per se*. Rather, the present study extends this literature by combining regional WPE abnormalities with the temporal persistence of coordinated complexity-drop cascades and a secondary network-level characterization of WPE-altered regions.

The reduced WPE observed in the inferior temporal and fusiform regions should be interpreted as a disease-related reduction in temporal flexibility rather than as healthy stabilization. In signal-complexity frameworks, lower entropy can indicate more stereotyped and less information-rich activity (Palencia, 2025). In AD, such reduction may arise from synaptic degeneration, impaired local circuit variability, reduced metabolic support, or altered neurovascular coupling. The inferior temporal gyrus and fusiform gyrus are central to high-order visual processing and are connected with memory-related and semantic systems (Brownsett and Wise, 2010; Guo et al., 2013; Rolls et al., 2024). Dysfunction in these regions could therefore contribute to visual recognition deficits, impaired object processing, visuospatial difficulties, and broader cognitive symptoms that extend beyond episodic memory impairment. The localization of reduced WPE to right inferior temporal and fusiform regions is notable because AD pathology is often discussed in relation to medial temporal, posterior cingulate, and default mode network regions (Mevel et al., 2011; Hafkemeijer et al., 2012). The present findings do not contradict that literature; instead, they expand it by suggesting that visual association cortex may show detectable abnormalities at the level of temporal signal complexity. Visual symptoms in AD are clinically relevant (Kiyosawa et al., 1989; Chang et al., 2014; Kaeser et al., 2015; Cerquera-Jaramillo et al., 2018), and dorsal and ventral visual stream dysfunction has been described in neuroimaging and neuropsychological studies. WPE may be sensitive to a different dimension of dysfunction than conventional connectivity or activation analyses, capturing local temporal pattern degradation even when standard measures do not fully describe regional impairment.

The functional connectivity findings provide a network-level characterization of the regions identified by the WPE analysis. Because seed selection and FC analysis were performed in the same sample, these connectivity findings should be interpreted as complementary network characterization rather than as statistically independent confirmation of the WPE abnormalities. Increased connectivity between WPE-altered visual association regions and dorsal visual pathway nodes indicates altered coupling between regions with abnormal local complexity and visuospatial/visuomotor systems. Previous studies have reported both increased connectivity and altered network segregation in AD, indicating that stronger functional coupling should not necessarily be interpreted as beneficial compensation (Ewers et al., 2021; Penalba-Sánchez et al., 2023; Chan et al., 2014). Therefore, the present data do not allow us to determine whether the observed connectivity increase is adaptive or maladaptive. Because the seeds were selected from WPE abnormalities identified in the same sample, the FC results should be regarded as secondary within-sample characterization; independent replication using externally defined or independently identified seeds will be important in future work.

Moreover, the temporal cascade findings suggest that AD may be associated with longer persistence of coordinated complexity reductions. In the primary analysis, longer Peak-to-End and Start-to-End intervals were observed in AD, and the same overall pattern was retained when the cascade criterion was varied to 8 or 12 simultaneously dropping regions. However, because no multiplicity correction was applied across the four temporal interval measures, these results should be interpreted as exploratory rather than confirmatory. Their biological significance also remains to be established through direct behavioral, physiological, longitudinal, and independent-sample validation.

Taken together, the reduced regional WPE, altered connectivity of WPE-defined regions, and prolonged complexity-drop cascades describe a convergent pattern of local, network-level, and temporal abnormalities in AD. These findings provide an integrative description of altered spontaneous brain dynamics rather than evidence for a specific causal mechanism. In particular, the present cross-sectional data cannot determine whether reduced local complexity precedes altered connectivity, whether increased connectivity represents compensation or dysfunction, or whether prolonged cascades are a cause or consequence of disease-related network changes.

From a clinical perspective, WPE provides information about regional temporal complexity that is complementary to conventional functional connectivity measures. The combination of regional WPE, connectivity characteristics, and cascade-duration metrics may therefore offer additional dimensions for characterizing AD-related functional abnormalities. However, whether these measures have diagnostic, prognostic, or treatment-monitoring value requires validation in larger longitudinal and independent cohorts.

This study has several limitations. First, the sample size was relatively limited, which may affect the statistical stability and generalizability of the whole-brain WPE findings. The primary Brainnetome ROI analysis used a stringent but uncorrected exploratory threshold; although the principal spatial pattern was reproduced with an alternative AAL parcellation under FDR correction, larger independent samples are required for confirmatory replication. Second, the cross-sectional design prevents us from determining whether reduced WPE, altered dorsal visual connectivity, and prolonged complexity-drop cascade dynamics represent disease-related deterioration or compensatory reorganization. Third, formal cognitive scale scores were not available for all healthy controls; these factors should be characterized in future cohorts. Fourth, the seed-based FC analysis used seed regions derived from WPE abnormalities identified in the same sample. Although WPE and FC represent different imaging measures, the seed selection and subsequent FC analysis were not statistically independent. Therefore, the FC findings should not be considered an independent validation of the WPE abnormalities. This data-driven seed selection may introduce selection dependence and could increase the apparent specificity of the observed FC differences. Accordingly, these results should be regarded as exploratory. Future studies should replicate the findings in independent cohorts or use split-sample, leave-one-out, or externally defined seed approaches to determine whether the observed FC alterations are robust and generalizable. Fifth, the four cascade-duration comparisons were not corrected for multiple testing and should be regarded as exploratory. Finally, the cascade-related measures are operational descriptions of WPE dynamics rather than direct neurophysiological states, and their biological significance requires further multimodal and longitudinal validation.

Despite these limitations, the study provides a complexity-based perspective on altered resting-state brain dynamics in AD. Reduced WPE in visual association regions, altered dorsal visual pathway connectivity, and prolonged complexity-drop cascade durations together indicate abnormalities in local signal complexity, network coupling, and temporal organization. This framework may help characterize multiple dimensions of spontaneous brain dysfunction in AD, although their underlying biological mechanisms require further validation.

## 5 Conclusion

Using resting-state fMRI and WPE, this study identified reduced signal complexity in the right inferior temporal and fusiform visual association regions in patients with AD. Seed-based connectivity analysis further revealed altered coupling between WPE-altered visual regions and dorsal visual pathway nodes, while temporal cascade analysis showed prolonged Peak-to-End and Start-to-End intervals. Together, these findings indicate that AD is associated with abnormalities in local temporal complexity, network connectivity, and the temporal persistence of complexity-drop cascades. WPE-based analysis may therefore provide a complementary framework for characterizing multidimensional alterations in spontaneous brain dynamics in AD, although the underlying biological significance requires further validation.

## Data Availability

The original contributions presented in this study are included in this article/Supplementary material, further inquiries can be directed to the corresponding authors.

## Footnotes

1
http://rfmri.org/dpabi
