# Supplementary material for: Reduced weighted permutation entropy in visual-temporal cortex and altered resting-state brain dynamics in Alzheimer’s disease
Source: Front Aging Neurosci. 2026 Oct 22;18:1921894. doi: 10.3389/fnagi.2026.1921894 (PMC13736609; doi:10.3389/fnagi.2026.1921894)
Supplement: Supplementary file 1 [file Data_Sheet_1.pdf]

Supplementary Results

S1:

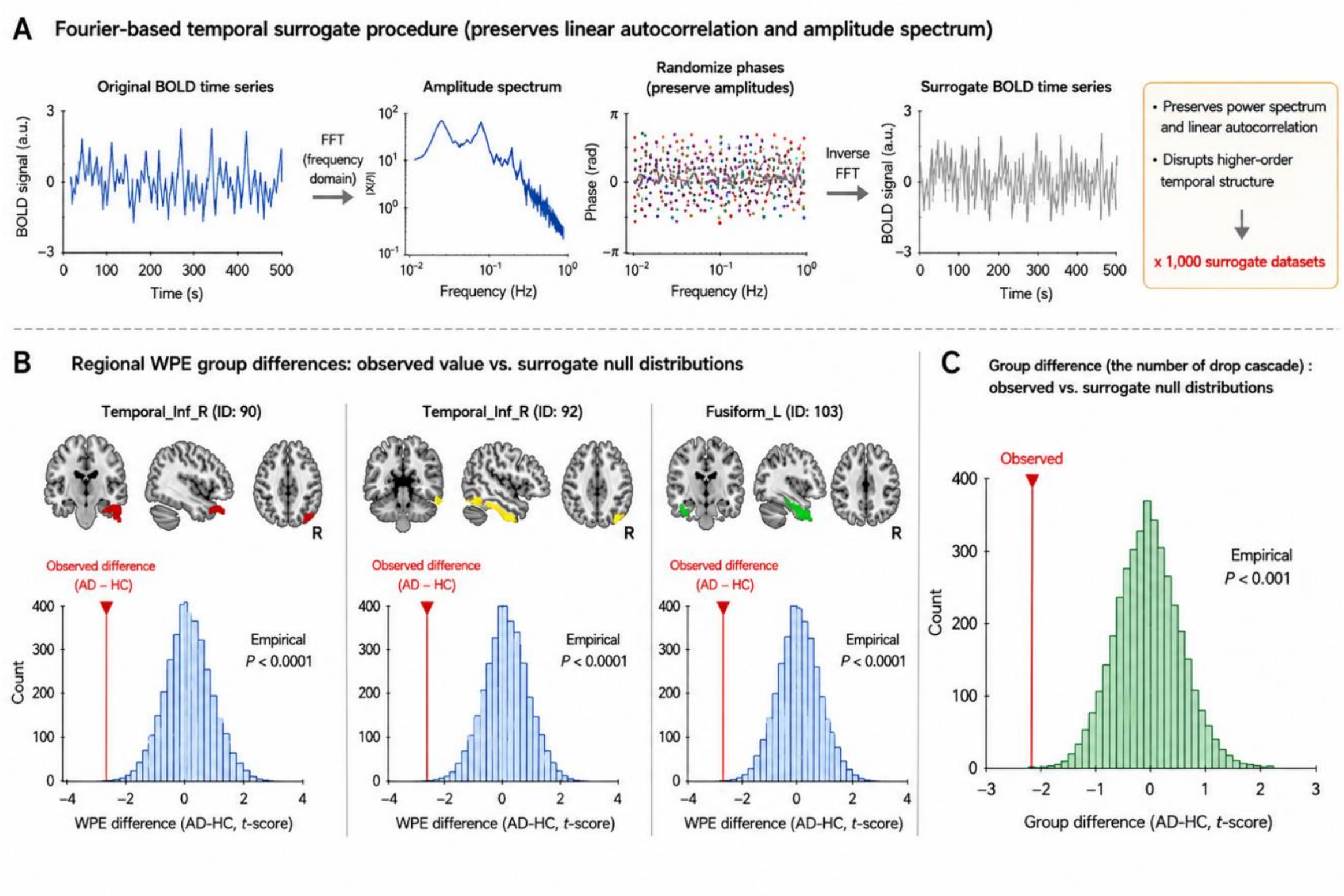

SFigure1. Surrogate validation of WPE abnormalities and drop-cascade dynamics. (A) Fourier-based surrogate procedure used to generate 1,000 surrogate BOLD datasets. (B) Empirical null distributions of regional WPE group differences. Red lines indicate the observed AD–HC differences (all empirical $P < 0.0001$). (C) Empirical null distribution of the AD–HC group difference in the number of drop cascades. The observed difference was significant (empirical $P < 0.001$).

S2:

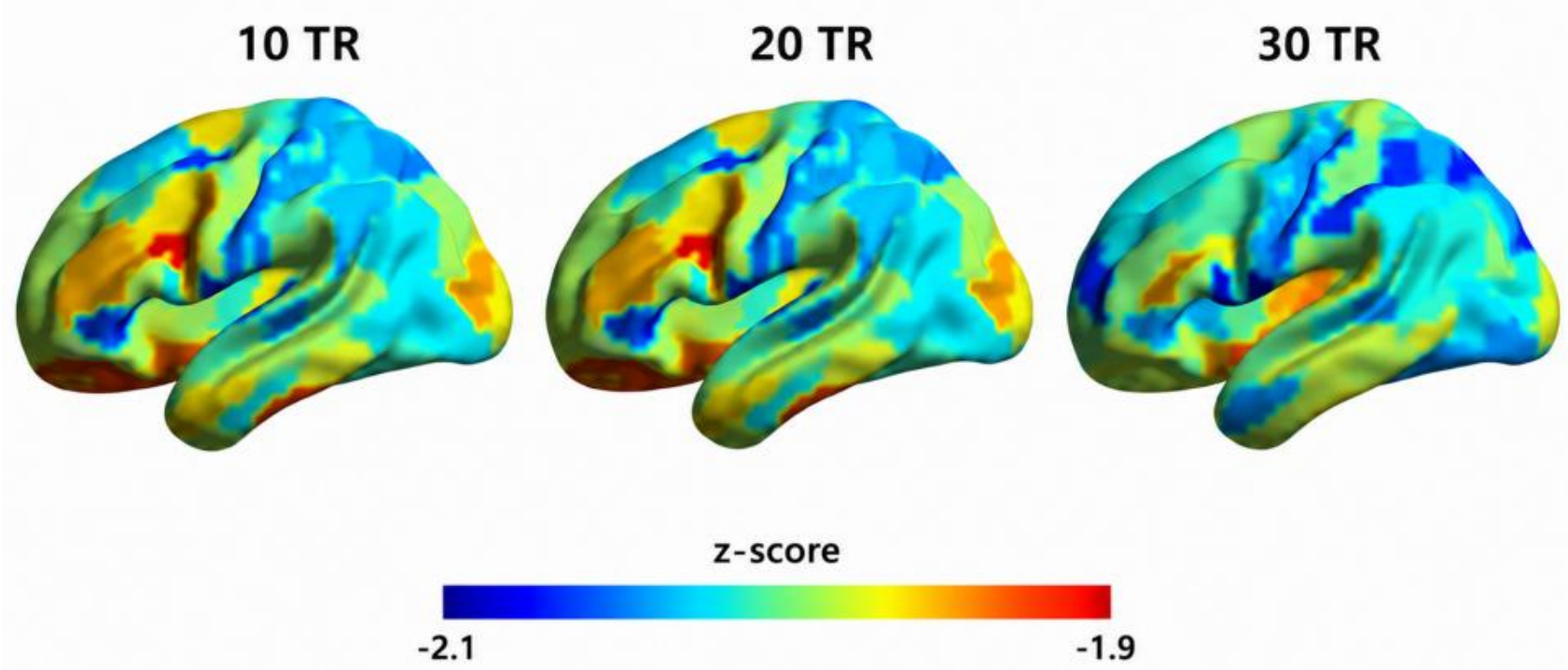

SFigure2. Spatial distribution of mean WPE across different window lengths. Mean whole-brain spatial distributions of weighted permutation entropy (WPE) across all participants are shown for three sliding-window lengths: 10 TR, 20 TR, and 30 TR. Brain surface maps are displayed on the left lateral view. The overall spatial pattern of WPE was broadly similar across window lengths, indicating that the large-scale distribution of WPE was relatively stable despite variation in the analysis window. The color bar represents z-score values ranging from -2.1 to -1.9.

S3

STable1: Demographic, clinical characteristics, and cognitive data of patients.

patient	Sex (1:male; 2female)	Age (year)	Education year	MMSE score	MoCA score	ADL score	Disease duration (year)	Medication	Daily dose
001	2	67	2	4	1	61	8	Memantine	20 mg/day
002	2	84	5	5	5	62	5	Memantine	20 mg/day
003	1	64	15	23	19	36	5	Memantine + Donepezil	10 mg/day + 10 mg/day
004	1	75	6	22	18	24	3	Donepezil	5 mg/day
005	2	57	12	4	2	37	6	Memantine	20 mg/day
006	1	79	9	16	7	47	2	Memantine + Donepezil	10 mg/day + 5 mg/day
007	1	66	14	6	6	48	5	Memantine	10 mg/day
008	2	63	7	1	1	67	6	Donepezil	5 mg/day
009	2	78	13	23	19	20	3	Donepezil	5 mg/day
010	2	78	8	15	16	36	3	Donepezil	10 mg/day
011	2	79	5	1	0	60	4	Memantine	10 mg/day
012	2	75	0	19	10	23	2	Memantine	5 mg/day
013	2	66	3	10	4	55	5	Meman	10 mg/day

								tine	
014	1	77	16	23	18	27	2	Memantine	10 mg/day
015	1	59	12	15	4	31	3	Donepezil	5 mg/day
016	2	65	14	21	15	21	2	Memantine	5 mg/day
017	2	55	5	7	3	33	5	Memantine + Donepezil	15 mg/day + 2.5 mg/day
018	2	69	8	22	13	39	3	Donepezil	10 mg/day
019	2	81	6	21	14	38	2	Memantine + Donepezil	5 mg/day + 5 mg/day
020	2	56	6	19	10	20	3	Not available	Not available

S4

STable2: Complexity abnormal brain regions of AD patients under different functional segmentation granularity.

Template	BAN (246 regions) (uncorrected $p<0.001$)	AAL (90 regions) (FDR corrected , $p<0.05$)
Decrease regions of AD patients (Label ID)	Tempora1_Inf_R(ID:90)	Temporal_Inf_R(90)
	($t=-3.62$, $p= 7.861*10^{-4}$)	($t=-3.91$, $p= 3.307*10^{-4}$)
	Tempora1_Inf_R(ID:92)	Temporal_Inf_R(90)
	($t=-3.56$, $p= 9.371*10^{-4}$)	($t=-3.91$, $p= 3.307*10^{-4}$)
	Fusiform_L (103)	Fusiform_L (55)
	($t=-3.60$, $p= 8.337*10^{-4}$)	$t=-4.13$, $p= 1.687*10^{-4}$)

S5

STable3: Sensitivity of Peak-to-End and Start-to-End group differences to alternative cascade-definition thresholds

Percentile threshold	Minimum brain regions	Measure	HC, mean ± SD	AD, mean ± SD	Hedges’ g	95% CI for g	Permutation p
1st (primary)	10	Peak-to-End	2.570 ± 0.730	3.173 ± 0.789	0.781	0.216–1.360	0.020
		Start-to-End	5.168 ± 1.076	6.030 ± 1.147	0.762	0.141–1.530	0.025
2nd	8	Peak-to-End	2.707 ± 0.728	3.368 ± 0.845	0.829	0.271 to 1.423	0.014
		Start-to-End	5.551 ± 1.201	6.411 ± 1.198	0.701	0.091 to 1.438	0.038
2nd	12	Peak-to-End	2.622 ± 0.752	3.220 ± 0.805	0.755	0.180 to 1.351	0.025
		Start-to-End	5.280 ± 1.066	6.268 ± 1.148	0.878	0.267 to 1.641	0.011
3rd	8	Peak-to-End	2.791 ± 0.754	3.398 ± 0.745	0.791	0.231 to 1.374	0.019
		Start-to-End	5.692 ± 1.105	6.707 ± 1.117	0.895	0.283 to 1.694	0.009
3rd	12	Peak-to-End	2.666 ± 0.730	3.349 ± 0.898	0.829	0.243 to 1.464	0.014
		Start-to-End	5.425 ± 1.089	6.318 ± 1.194	0.770	0.153 to 1.519	0.023

The primary cascade definition used the 1st percentile and a minimum of 10 simultaneously involved regions. Alternative sensitivity-analysis definitions included the 2nd and 3rd percentile thresholds combined with minimum regional extents of 8 and 12 regions. Group differences were assessed using two-sided nonparametric permutation tests. Effect sizes are reported as Hedges’ g, with 95% confidence intervals estimated using bootstrap resampling. Positive Hedges’ g values indicate longer cascade-duration measures in the AD group than in the HC group. HC, healthy controls; AD, Alzheimer’s disease; CI, confidence interval.
